# V-Phaser 2: variant inference for viral populations

**DOI:** 10.1186/1471-2164-14-674

**Published:** 2013-10-03

**Authors:** Xiao Yang, Patrick Charlebois, Alex Macalalad, Matthew R Henn, Michael C Zody

**Affiliations:** 1 Broad Institute of MIT & Harvard, 7 Cambridge Center, Cambridge, MA 02142 USA

**Keywords:** Viral population, Variant calling, Length polymorphisms, Phasing, Next generation sequencing

## Abstract

**Background:**

Massively parallel sequencing offers the possibility of revolutionizing the study of viral populations by providing ultra deep sequencing (tens to hundreds of thousand fold coverage) of complete viral genomes. However, differentiation of true low frequency variants from sequencing errors remains challenging.

**Results:**

We developed a software package, V-Phaser 2, for inferring intrahost diversity within viral populations. This program adds three major new methodologies to the state of the art: a technique to efficiently utilize paired end read data for calling phased variants, a new strategy to represent and infer length polymorphisms, and an in line filter for erroneous calls arising from systematic sequencing artifacts. We have also heavily optimized memory and run time performance. This combination of algorithmic and technical advances allows V-Phaser 2 to fully utilize extremely deep paired end sequencing data (such as generated by Illumina sequencers) to accurately infer low frequency intrahost variants in viral populations in reasonable time on a standard desktop computer. V-Phaser 2 was validated and compared to both QuRe and the original V-Phaser on three datasets obtained from two viral populations: a mixture of eight known strains of West Nile Virus (WNV) sequenced on both 454 Titanium and Illumina MiSeq and a mixture of twenty-four known strains of WNV sequenced only on 454 Titanium. V-Phaser 2 outperformed the other two programs in both sensitivity and specificity while using more than five fold less time and memory.

**Conclusions:**

We developed V-Phaser 2, a publicly available software tool (V-Phaser 2 can be accessed via: http://www.broadinstitute.org/scientific-community/science/projects/viral-genomics/v-phaser-2 and is freely available for academic use) that enables the efficient analysis of ultra-deep sequencing data produced by common next generation sequencing platforms for viral populations.

## Background

Inferring variants for viral populations is crucial for understanding disease progression, determining the effect of immune pressure on viral genotype, optimizing vaccine design, and identifying and detecting drug resistance mutations [1-5].

The basic steps of variant inference in viral populations start by aligning reads to a reference genome, either previously assembled [6,7] or assembled *de novo*[4,8]. Assembling each virus *de novo* prior to variant calling is advantageous as the sample consensus may be highly diverged from any existing reference (if a reference even exists). Also, aligning to a reference that differs too much from the reads may result in reference bias in variant calling or spurious variant calling due to poor alignments [4]. Then, ideally, any base that differs from the reference base shall be a variant. However, a base difference may occur due to sequencing errors and hence a variant can be identified if it appears more frequent than sequencing errors [9-11]. This is typically referred to as a pileup model. To identify variants with much lower frequency, phylogenetic relationships among multiple sites, termed *phasing*, need to be considered [12]. The rationale is that errors typically appear more randomly and less concordantly with each other compared to real variants that are phylogenetically related, *i.e.* in phase.

Because of the utilization of the phasing model, V-Phaser [12] fares better in variant calling compared to other programs for viral population. It was mainly applied to 454 sequencing data, which typically has a few hundred fold read coverage. Illumina sequencing is a cost-effective alternative compared to 454 sequencing and it has several advantages: Illumina data typically provide thousands to tens of thousands read coverage, with which low frequency variants are more likely to be captured. The dominant error mode in 454 data is insertion/deletion error caused by incorrect counting of homopolymers and associated substitutions resulting from carry forward and incomplete extension (CAFIE). In contrast, Illumina errors are primarily single base substitutions. The former results in spurious frameshifts in coding regions and also introduces spurious length polymorphisms (LPs, or indels), which are typically more difficult to manage compared to spurious single nucleotide polymorphisms (SNPs). However, when applied to Illumina sequencing data, V-Phaser has poor scalability. In addition, it is not able to directly utilize phasing information provided by paired end reads.

We developed the V-Phaser 2 program that overcomes these limitations of V-Phaser [12]. V-Phaser 2 utilizes paired reads in phasing, extending the distance between phased sites from a read length to a fragment length. A more efficient implementation of the base quality recalibration and error inference algorithms vastly reduces run time and memory use, making it possible to analyze much deeper sequencing data. In addition, in V-Phaser 2, we further addressed the following general issues in the existing viral variant calling methods: 

1) Variant inference programs typically infer variants with respect to a given reference. However, the reference genome may contain bases that do not represent the majority of the read data. This may result in extra computation and neglecting of real variants in phase. We alleviate this issue by first *de novo* assembling the data and creating the reference to which reads can be realigned [4,8]. Then we recompute the consensus using alignment information alone to further avoid the misrepresentation of the consensus during variant calling.

2) The representation of LPs is not standardized, and the previous methods have been mostly focused on SNPs. We introduced a method to represent and infer LPs.

3) Alignment programs may have difficulties generating accurate alignments in homopolymeric regions and towards the ends of reads. These alignment artifacts may not be avoided. Therefore, we integrated a filtering strategy that can be used to remove probable recurrent or correlated artifacts based on strand bias.

We demonstrate the effectiveness of V-Phaser 2 on a mixed population of eight known West Nile virus (WNV) strains, sequenced by both 454 and Illumina MiSeq for 900 fold and 4500 fold effective coverage, respectively, and on a more complex sample consisting of twenty-four WNV strains sequenced by 454 with an effective coverage of around 1,000 fold. V-Phaser 2 has comparable sensitivity to V-Phaser but is superior in controlling false positives. It reduces compute-time and memory usage substantially over V-Phaser. When compared to relevant viral variant inference programs like QuRe [10], V-Phaser 2 has a higher sensitivity and achieved better run-time and memory usage as well.

## Methods

V-Phaser 2 requires only read alignment in BAM format [13] as the input. For each reference genome in the BAM file, it reports both single nucleotide polymorphisms (SNPs) and length polymorphsms (LPs). The API of Bamtools [14] was used for accessing the BAM file. To be precise about terminology, we term any base that differs from the consensus base (typically the base in the reference genome) a *single nucleotide difference* (SND). When a SND is statistically validated, it is termed a *single nucleotide variant* (SNV). We further term the corresponding consensus a *single nucleotide consensus* (SNC). Likewise, as length polymorphism occurs when an oligo is inserted or deleted compared to the reference, we use the terms *length polymorphism difference* (LPD), *length polymorphism variant* (LPV) and *length polymorphism consensus* (LPC) to denote the inserted or deleted oligo, a statistically validated LPD, and the corresponding consensus.

The basic idea and strategy used in V-Phaser 2 are outlined below.

**Figure 1:**
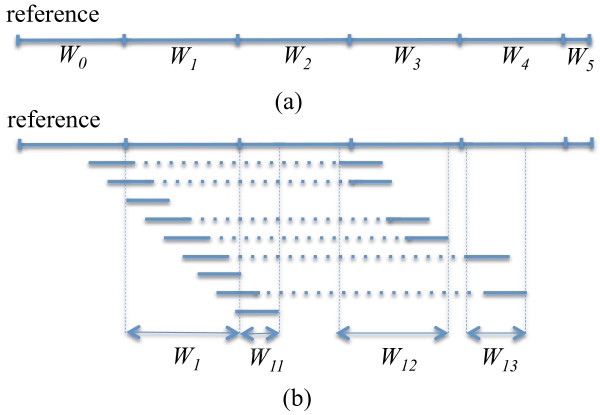
**Reduce memory footprint by genome partitioning and analysis. (a)** The reference genome, denoted by a horizontal line, is partitioned into 6 non-overlapping windows *W*_*i*_s (0≤*i*≤5). **(b)** When analyzing each window, all reads overlapping this window as well as the relevant paired end reads are considered. Each read (denoted by a short line) is placed underneath the location where it is aligned to the reference. Each read pair is connected by a dotted line. Assuming *W*_1_ is the target window, all reads overlapping with *W*_1_ will be considered for pileup and phase analysis. In addition, reads overlapping with *W*_1*i*_s, 1≤*i*≤3, will be considered for phase analysis.

To be able to handle ultra-deep coverage data, *e.g.* >3,000 fold, using a moderate amount of memory, we process each reference genome in the BAM file by first partitioning it into a set of non-overlapping target windows with equal length except the last one. An example is shown in Figure 1 (a), where the reference is divided into 6 target windows. Then, these target windows are processed in 5 ^′^ to 3 ^′^ direction.

For each target window, we obtain complete read alignment information by analyzing any read (partially or fully) aligned to this window. For example, in Figure 1 (b), let *W*_1_ be the target and **R**_1_ be the set of all reads aligned to *W*_1_. Then, we infer for every alignment column *c* in *W*_1_ variants that are statistically significant given base error probabilities in *c* (see details later). To infer variants that may be in phase with variants in *c*, we further investigate alignment information provided by any read that is paired with some read in **R**_1_. For *W*_1_, this involves reads in windows *W*_12_ and *W*_13_ in Figure 1 (b). Thus, a phased variant may belong to an alignment column *c*^′^ that is either contained by *W*_1_, *W*_11_, *W*_12_ or *W*_13_. Note that *c*^′^ may involve only a subset of reads aligning to this column in the BAM file, *e.g.* in *W*_12_. We calculate the mean and standard deviation of fragment size in the BAM file and allow users to limit the distance between paired reads to be considered for phasing. Like V-Phaser [12], we consider no more than two columns for phasing, as increasing this number may not necessarily improve the results nor be computationally practical.

V-Phaser 2 combines the strategies used in GATK [15] and V-Phaser to calibrate sequencing error probability. As in GATK, sequencing error probability is determined by a set of joint variables (e.g., read cycle, quality score, etc.). However, unlike both GATK and V-Phaser, V-Phaser 2 no longer outputs re-calibrated quality scores. Instead, error probabilities are directly calculated by dividing the observed sequencing errors by total number of observations defined by the joint variables. Calculated error probabilities are then used during variant inference rather than drawn from quality scores (see details later). Observed sequencing errors are initialized to be all differences between the reads and the consensus, as it is typically too costly to add a known control sequence, as used in GATK, to be sequenced along with the viral sample for the purpose of measuring sequencing errors.

**Figure 2:**
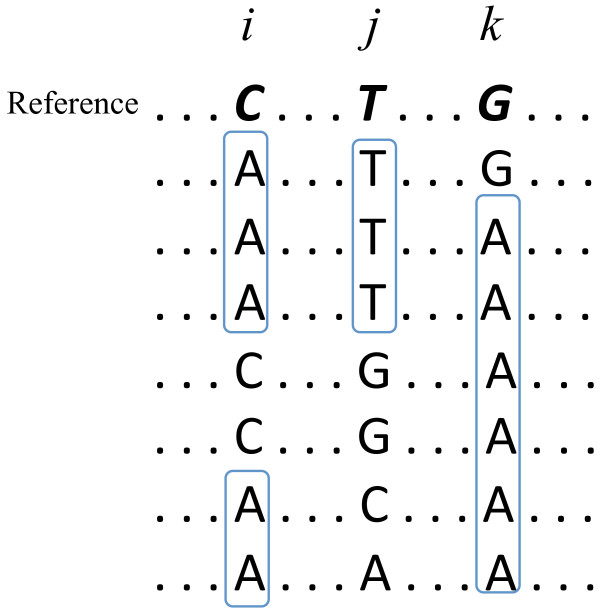
**An example of read alignment with respect to a reference, where only three positions are shown.** Reference bases are bolded. The dominant base in each alignment column is circled. Recalculation of consensus base may fix reference base, initially used for read alignment.

We differentiate error probabilities of LPDs and SNDs; for major NGS platforms indel error rate differs substantially from substitution error rate. Note that we do not differentiate insertion from deletion LPDs as in the current application, this classification is only relative to the chosen reference genome to which the reads were aligned. Furthermore, as compared to most existing methods that assume the reference base to be the correct consensus, we do not require the knowledge of the reference genome(s) based on which the BAM file was generated. Instead, the consensus is recalculated using read alignment information, and when multiple bases occur at the same frequency, the alphabetically smallest one is chosen to be the consensus. By doing so, we avoid the unnecessary variant inference and inspect co-variants that would be neglected otherwise. For example, in Figure 2, after consensus recalculation, base “A” (column *k*) will not be reported as a variant and the co-variation of “CG” (columns *i* and *j*) becomes evident.

More importantly, recalculated consensus bases using read alignment can better serve as the back-bone sequence of the underlying population, based on which SNVs and LPVs are inferred.

Given the calibrated error probabilities, V-Phaser 2 iterates through the following steps until no further variants are inferred: 1) calculate SND and LPD error probabilities from the data, 2) infer SNVs and LPVs, 3) remove any alignment column in which a variant has been inferred from error probability calculation. In the second step, the pileup model is first used to infer variants for single alignment column followed by phasing model, where the already inferred variants would not be considered. Lastly, inferred variants that show strand-bias are removed. Below, we present the details of the method.

### Calibrating sequencing error probability

We use substitution and indel error probabilities to infer SNVs and LPVs, respectively. Since an error base is identified by comparing with the consensus, we first discuss consensus base recalculation using read alignment information. For ease of presentation, we assume reads are aligned in the forward direction. The methodology remains the same for reversely mapped reads with technical differences only.

#### Consensus base recalculation

**Figure 3:**
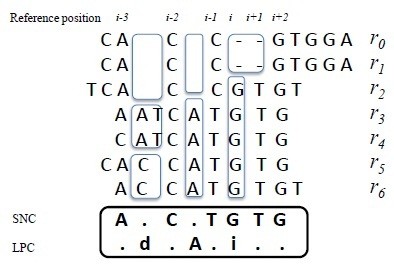
**An illustration of consensus base recalculation between reference positions (*i *−3) and (*i *+2) using read alignment information.** The alignment of 7 reads (*r*_0_,*r*_1_,…,*r*_6_) with respect to the reference genomic positions is given. *c*_*i*−3,*i*−2_ and *c*_*i*−2,*i*−1_ denote insertions. Deletions are denoted by “-”s. Each type of length polymorphism is circulated by a blank rectangular. Recalculated single nucleotide consensus (SNC) and length polymorphic consensus (LPC) are shown below the alignment, where each dot denotes that the corresponding consensus base will not be considered.

Let *c*_*i*_ denote an alignment column with respect to the reference position *i*, and *c*_*i*,*i*+1_ denote an inserted column between positions *i* and *i*+1. We classify an alignment column to be 1) an inserted column, 2) a column containing deletion start sites among a subset of reads, or 3) neither 1) nor 2). For example, in Figure 3, *c*_*i*−3,*i*−2_ is type 1, *c*_*i*_ is type 2, and *c*_*i*+2_ is type 3.

For type 1 column, we calculate LPC only. Each type of insertion is considered as a “base” in the column. For instance, column *c*_*i*−3,*i*−2_ contains three types of bases: an null base which we denote as “d” (stands for deletion with respect to the reference), an inserted base of “AT”, and an inserted base of “C”. “d” is selected to be the LPC as it is the most frequent type.

For type 2 column, we calculate both SNC and LPC. A deletion as a whole is treated as a LPD, thus, we consider only deletions starting at this alignment column. Since the deleted fragment is unknown, we treat deletions with the same length as one type of base. Deletions of length zero is denoted as “i” (stands for insertion with respect to the reference). For instance, in Figure 3, column *c*_*i*_ contains two types of deletions: “D2”, a length 2 deletion, and “i”, where the latter is the LPC. We further calculate SNC by neglecting all deletions in the alignment column, using the same method as in type 3 below.

For type 3 column, neither insertions nor the start of deletions occur, we calculate SNC only, which is the dominant nucleotide. For instance, the consensus base is “G” for column *c*_*i*+2_ in Figure 3. Any base that failed to be called by the sequencer, typically marked as an “N” in a read, is neglected.

The aforementioned consensus calculation is naturally extended to the phasing stage, where the phasing consensus is derived by concatenating the consensus of two alignment columns of interest. At most four types of phasing consensus would be calculated.

#### Error probability calculation

We associate a specific base with the following variables: read cycle (denoted by *α*), di-nucleotide content (denoted by *β*), quality score (denoted by *γ*), and the order of the read in the mate pair (denoted by *θ*). Let *C*_·_ (·∈{*α*,*β*,*γ*,*θ*}) denote the cardinality of the corresponding variable, which is uniquely determined when an input dataset is specified. Then the total combinations is given by *C*_*α*_×*C*_*β*_×*C*_*γ*_×*C*_*θ*_. For example, assuming in a Illumina paired read dataset, the maximum read cycle is 101, the total number of di-nucleotides is 4^2^=16, the quality score value is in the range of ‘#’ and ‘I’ (39 ASCII characters), and a read can either be the first or the second in the mate-pair. Then *C*_*α*_=101,*C*_*β*_=16,*C*_*γ*_=39 and *C*_*θ*_=2, and the total number of such combinations is *C*_*α*_×*C*_*β*_×*C*_*γ*_×*C*_*θ*_=126,048.

A base can be uniquely projected to one of these combinations, which we term as a *bucket* for the base, and the index of the bucket can be calculated as *α*×*C*_*β*_×*C*_*γ*_×*C*_*θ*_+*β*×*C*_*γ*_×*C*_*θ*_+*γ*×*C*_*θ*_+*θ*. Note that *β*, *γ* and *θ* are converted to the integer values.

Given the read data, two values are computed for each bucket: the frequency of total bases in the bucket, *N*_*all*_, and the frequency of bases that do not match the recalculated consensus bases in their corresponding alignment columns, *N*_*mis*_. Then, the error probability of each bucket is calculated as *N*_*mis*_/*N*_*all*_. This may be an over estimation of error probability for some buckets when the mismatches resulted from real variants in the data are included.

For a nucleotide in the alignment, the calculation of its bucket is straight-forward. For an insertion or a deletion, *α* and *γ* are assigned the same values as the nucleotide preceding it in the 5 ^′^ region on the same read; and *β* is determined by the di-nucleotide that is formed by concatenating its two neighboring nucleotides. For example, in Figure 3, *β*=1 and the di-nucleotide is “AC” for the “AT” insertion of read *r*_3_, and *β*=3, and the di-nucleotide is “CG” for the 2 base deletion of read *r*_0_.

Using the above method, we create buckets for calculating SND and LPD error probabilities, respectively. For the former, all bases in the type 3 columns and all nucleotide bases in type 2 columns are used for calculating two bucket values, where SNC is used to determine if a mismatch occurs; and for the latter, all bases in every column are used for calculation, except that LPC is used to determine mismatches. Thus, given a base in the alignment, let it be a nucleotide, a deletion, or an insertion, the error probability can be calculated by first identifying its bucket and then by dividing the two values in the bucket as described above. The phasing error probability for two columns of interest is equal to the product of the LPD or SND error probabilities of both columns; hence, up to four types of phasing probability are calculated: SND versus SND, SND versus LPD, LPD versus SND, and LPD versus LPD.

To provide flexibility for different applications, we allow users to use a subset of these variables. For example, when the target viral genomic region of interest is fully contained by every read of the input, there exists strong correlation between both cycle in the read and dinucleotide content and sites of real variation. In such cases, it is more appropriate to neglect these variables in the model. When none of these variables are used, the error probability becomes independently and identically distributed, which is equivalent to dividing the total number of non-consensus bases by the total number of bases in the alignment data.

### Inferring SNVs and LPVs

The solution boils down to answering the following two questions: 1) given an alignment column and the error probability for each base in this column, does any non-consensus base occur more frequently than expected due to sequencing errors, and 2) given two alignment columns and the error probability for each base involved, does any pair of non-consensus bases co-occur more frequently than expected due to sequencing errors.

**Figure 4:**
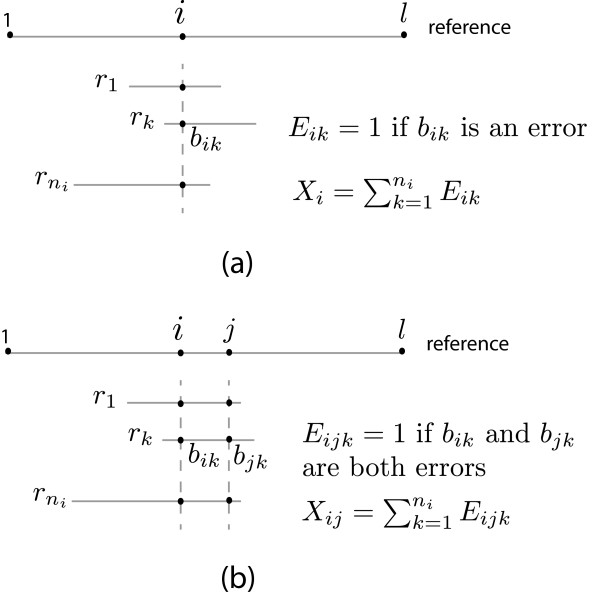
**Error model for inferring statistically significant variants based on (a) Pileup and (b) Phasing.** The reference genome has length *l*. *b*_*ik*_ is the *i*^*th*^ base of read *r*_*k*_.

We first illustrate the pileup and phasing probability model defined by [12] in Figure 4. $\left\{r_{1},r_{2},\ldots ,r_{n_{i}}\right\}$ are the reads overlapping reference position *i* in pileup (a) or both *i* and *j* in phasing (b) models. Base *b*_*ik*_ having error probability *p*_*ik*_ is considered an error if it differs from the consensus. The corresponding indicator functions *E*_*ik*_=1 when *b*_*ik*_ is an error and *E*_*ijk*_=1 when both *b*_*ik*_ and *b*_*jk*_ are errors. The former two questions are then answered by determining if the following two functions are statistically significant: $P(X_{i}=\overset{n_{i}}{\underset{k=1}{\sum }}E_{\text{ij}}\geq x)=\overset{n_{i}}{\underset{m=x}{\sum }}P_{m}(n_{i})$ and $P(X_{\text{ij}}=\overset{n_{i}}{\underset{k=1}{\sum }}E_{\text{ijk}}\geq x)=\overset{n_{i}}{\underset{m=x}{\sum }}P_{m}(n_{i})$, where *P*_*m*_(*n*_*i*_) denote the probability that given coverage *n*_*i*_ at position *i*, the probability of observing *m* errors.

As the error probability of each base may differ, *P*_*m*_(*n*_*i*_) follows the Poisson binomial distribution and can be calculated exactly by a recursive function in $O(n_{i}^{2})$ time [16]. This strategy was used in V-Phaser. However, as in the current application *n*_*i*_ is typically large and the error probability is small, this can be well approximated by the Poisson distribution *P*_*m*_(*n*_*i*_)≈*Poisson*(*m*;*λ*) [16], where $\lambda =\overset{n_{i}}{\underset{k=1}{\sum }}p_{\text{ik}}$ for the pileup model and $\lambda =\overset{n_{i}}{\underset{k=1}{\sum }}p_{\text{ik}}p_{\text{jk}}$ for the phasing model. Use of this approximation makes the run time linear with respect to the coverage and results in substantial speed up for relevant coverages.

Using the above strategy, we inspect for every single alignment column the probability of observing SNDs and LPDs and for each pair of alignment columns all four combinations of probabilities of observing phased SNDs and LPDs. Šidák correction [17] was used to correct for multiple tests.

### Filtering systematic artifacts

The statistical methods used to distinguish real variants from sequencing error assume error modes that follow the models above. In practice, some sequencing errors systematically occur at certain loci on certain instruments [18]. Many such artifacts display a strong bias towards one strand of sequencing, making strand symmetry of the alleles a simple and useful filter [15]. Hence, we applied either a Chi-square test or Fisher’s exact test to each identified variant by generating a two by two table, where the rows are labeled as the forward or the reverse strand, and the columns are labeled as the target allele and other alleles, and each entry of the cell registers the corresponding count. Chi-square test was applied whenever all cell entries in the table have an expected value of ≥5, otherwise, Fisher’s exact test was used. To correct for multiple hypothesis testing, we used the Benjamini - Hochberg procedure [19] to control for false discovery rate (FDR) at the level of 0.05. Note that although the above procedure may be effective in removing spurious variants, there is a risk of removing real variants and the reason is not yet clear.

### Generation of West Nile virus sequence data

Sequence data for evaluation was generated as described in Macalalad et al. [12]. Briefly eight (8-mix) or twenty-four (24-mix) strains of West Nile virus (WNV) isolated from birds and mosquitos were pooled at equal concentration and used to infect C6/36 cells. After competitive replication, the viral RNA was isolated, reverse transcribed to cDNA, and then amplified using four overlapping amplicons each of approximately 3 kb length. The resulting amplicons were used as input to library construction for 454 and Illumina sequencing.

## Result and discussion

**Figure 5:**
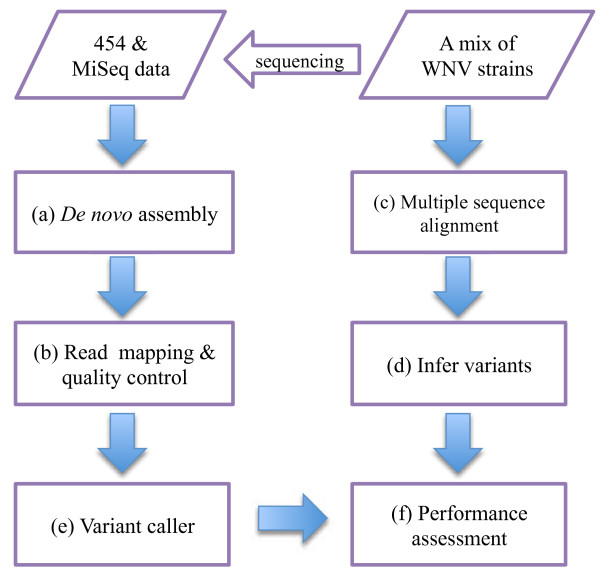
**Variant validation.** Variants inferred by a variant calling program on the sequence data of a mixed population sample (left panel) are validated against real variants, which are inferred to be bases at positions of the multiple sequence alignment of the parental strains of the same sample, where not all bases are identical (right panel).

To validate the result of variant inference, we used a sample consisting of a mixture of 8 known strains of West Nile Virus, sequenced by 454 [12] and Illumina MiSeq sequencers, respectively. A more complex sample consisting of a mixture of 24 known strains of West Nile Virus sequenced by 454 was also analyzed. The workflow of our validation process is given in Figure 5, where each part of the diagram is described in detail below.

### Data sets, assembly and read mapping

The input datasets (Table 1) were first assembled *de novo* using AV454 [4] to generate reference genomes representing the underlying populations (Figure 5 (a)). The resulting reference genomes are 10,621bp and 10,664bp in length for the 8-mix and the 24-mix samples, respectively.

**Table 1:** Dataset description and read mapping results

	**Data**	**# of**	**% reads**	**Coverage**
		**reads**	**mapped**	
	454	26,771	99.9	919
8-mix	MiSeq	308,260	69.1	4,512
	MiSeq trimmed	290,875	94.4	4,466
24-mix	454	39,759	95.7	1,074

Next, the 454 reads and MiSeq reads were mapped to the corresponding reference genomes (Figure 5 (b)) using Mosaik 2.1.33 (http://code.google.com/p/mosaik-aligner/, with parameters “m = all, gop = 15, hgop = 4, gep = 6.7, mmp = 0.15, minp = 0.5” for 454 data and “m = all, gop = 50, hgop = 40, gep = 15, mmp = 0.07, minp = 0.9” for Illumina data). RC454 [4] was further applied to correct homopolymer and carry-forward errors in 454 data. The mapping results (Table 1) indicate that the 454 data has high quality whereas the quality of MiSeq data for the 8-mix is relatively poor. As the quality of read mapping may affect variant inference substantially, trimming is applied to MiSeq data: since the bad quality suffix of a read is indicated by a quality score of 2, we trim MiSeq reads by removing 3^′^ suffix of each read with such a quality, and retain only reads with a minimum length of 30bp. Post-trimming, the percentage of mapped reads increased to 94.4%, indicating a substantial improvement of read quality. Lastly, we use GATK (version 2.1-13-g1706365) indel realigner component [15] to adjust the local alignment of indels for MiSeq read mapping results.

### Benchmark variants in the mixed populations

In order to evaluate the result of variant inference, we need to determine real variants in the mixed population, which can be done by inspecting the known parental viral strains in the mixed population.

Multiple sequence alignment of all known strains and the assembled reference genome was obtained (Figure 5 (c)). Any alignment position containing more than one type of base is considered to have variation. This way we obtained 121 SNVs and no LPV for the 8-mix data and 258 SNVs and 3 LPVs for the 24-mix data. We need to mention that for the 24-mix data, we only successfully obtained 21 individual strains in the mix whereas the remaining 3 failed the sequencing. Thus, it is likely that the 24-mix sample contains very low quantity of viral particles for the 3 strains that failed sequencing process. Hence, the multiple sequence alignment was performed on the 21 input strains and the assembled sequence.

Some of the variants in the input strains may not necessarily be observed in the sequencing data. After eliminating those that have 0 instances in the 454 and MiSeq data for the 8-mix and in the 454 data for the 24-mix, we obtained 114 variants for the former and 243 variants for the latter as our benchmark (Figure 5 (d)). All of these variants are SNVs. The frequencies of these variants in the sequence data are summarized in Table 2. Note that it is challenging to determine the origin of additional variants that are not present in the sequences of the input strains but which may appear at high confidence in the data. First, as we have only consensus sequences of the parental strains, some may be low frequency variants in the input strains that are detected in the final mix but not observed in the alignment of parental consensus. For the 24-mix, these may also come from the parental strains which failed consensus sequencing. Some may also represent real viral variants occurring in cell culture during the competitive growth of the mixed strains. Finally, some may result from errors occurring during reverse transcription or early rounds of PCR amplification that are at detectable frequency but represent true reads by the sequencing instrument and so are not detectable as errors under the model used even though they do not represent true variants in the input RNA. Because these were not validated by experiments, we choose not to explore them for benchmarking and instead treat them all as false positives despite the fact that some may be real variants.

**Table 2:** The variants are divided into four different bins according to their observed frequencies in the read alignment data

**Data**		**Number of variants with frequency**			
		**(0, 0.5%]**	**(0.5%, 1%]**	**(1%, 5%]**	**(5%, 50%)**
8-mix	454	3	9	11	91
	MiSeq	3	10	6	95
24-mix	454	4	11	148	80

In each set of sequencing data, we examine only those positions where multiple base types are observed in the raw alignments. As a result, a total of 924, 9,714, and 3,134 positions are inspected in the 8-mix 454 data, 8-mix Illumina MiSeq data, and 24-mix 454 data, respectively (Table 3). The remaining sites contain no non-consensus calls are trivially called as consensus without need for application of statistical inference.

**Table 3:** Variant inference results of V-Phaser, QuRe and V-Phaser 2 on three datasets

**Data**		**Method**	**TP**	**FP**	**FN**	**TN**	**Sensitivity**	**Specificity**	**Run time (min)**	**Memory (G)**
		V-Phaser	110	116	4	694	**96.49%**	85.56%	34.3	12.51
	454	QuRe	59	19	55	791	51.75%	**97.65%**	6.5	7.50
		V-Phaser 2	105	27	9	783	92.11%	96.67%	**0.9**	**0.04**
8-mix	Illumina	V-Phaser	-	-	-	-	-	-	>600.0	>100.00
	MiSeq	QuRe	87	84	27	9,516	76.31%	99.13%	206.3	11.00
		V-Phaser 2	106	40	8	9,560	**92.98%**	**99.58%**	**36.1**	**0.73**
		V-Phaser	194	180	49	2,711	79.84%	93.84%	120.6	18.20
24-mix	454	QuRe	124	201	119	2,690	51.03%	93.05%	19.5	7.80
		V-Phaser 2	196	61	48	2,829	**80.33%**	**97.89%**	**2.4**	**0.14**

The results show that V-Phaser 2 substantially reduces the run-time and memory usage compared to V-Phaser and QuRe; V-Phaser 2 has comparable sensitivity with V-Phaser, where both are better than QuRe; V-Phaser 2 has comparable specificity compared to QuRe, where both are better than V-Phaser. ‘-’ indicates the corresponding value was not measured. We terminated V-Phaser after it uses over 100G memory on the Illumina MiSeq data. For each performance measure, the best value is bolded.

### Comparing V-Phaser 2 with V-Phaser and relevant programs

In Table 3, we present the variant inference results of V-Phaser 2, V-Phaser, and QuRe [10] on all three datasets with the default parameters for all. Attempts have been made to test all programs reviewed in [20], nonetheless, only QuRe successfully ran on all the datasets. Although another program, Segminator II [11], can handle all three datasets as well, we consider the comparison would not be meaningful as in deep coverage data, it reports variants in every single position with respect to the reference genome and the burden of choosing the correct variants is left to the user. QuRe reported haplotypes for the underlying population, where multiple sequence alignment of the haplotypes were created using MUSCLE and the variants were determined at positions wherever variations occur. The coordinates of these variants were then transformed to the coordinates of the corresponding reference genome to be comparable.

All of the experiments were performed on a Linux system, with 24 heterogeneous AMD Opteron Processors working at 800 MHz and 2400 MHz. V-Phaser used one core, whereas both V-Phaser 2 and QuRe can take advantage of multi-core architecture, where eight cores were used.

For all three datasets, V-Phaser 2 is the most efficient. It achieved 38-50 fold reduction in run-time and 130-320 fold reduction in memory usage when compared to V-Phaser for runs where V-Phaser was able to complete. For the 8-mix Illumina MiSeq data, we terminated V-Phaser after it used exceedingly large memory (over 100 Gb). V-Phaser 2 is also substantially more efficient compared to QuRe, where 7-8 fold reduction in run-time and 15-187 fold reduction in memory usage were observed.

V-Phaser 2 and V-Phaser have comparable sensitivity, where both outperform QuRe large as a result of their utilization of the phasing model. More specifically, for the 8-mix 454 data, V-Phaser 2 inferred 105 real variants that are fully contained in the variant set inferred by V-Phaser. All three real variants with frequency ≤0.5*%* (Table 2) were missed by both programs. V-Phaser inferred five more true variants than V-Phaser 2, where four of them have frequency ≤1.6*%* and the remaining one has frequency 12.08% but showed strand bias. QuRe misses about half of the real variants, and the minimum frequency of the variants identified is 5.79%. For the 8-mix MiSeq data, the sensitivity of QuRe improved on the same sample but still trailed V-Phaser 2, which has comparable sensitivity to the result it produced for the 454 data. It is worth noting that although the same 8-mix sample is sequenced by 454 and Illumina MiSeq, the variant calling results from V-Phaser 2 and QuRe differ, mainly because of the differences in sequencing depth and read alignment. For the 24-mix 454 data, the coverage is slightly higher compared to the 454 data of the 8-mix (Table 1). V-Phaser and V-Phaser 2 inferred 183 real variants in common, and both missed a common set of 34 real variants. The uniquely inferred real variants are 11 for V-Phaser and 13 for V-Phaser 2. The high percentage of overlap in inferred real variants for both 454 datasets indicates that the two programs are highly consistent.

In general, V-Phaser 2 has better specificity compared to V-Phaser, which is due to the inclusion of the strand bias test in the former that eliminated many false positive inferences. It also inferred many fewer false positives compared to QuRe on two of the datasets. As we discussed earlier, the homopolymer sequencing errors are more difficult to handle. This has been reflected in the results of QuRe, which infers many false LPVs in the 454 data but none in the MiSeq data.

Although in the current datasets, there should be no real LPVs based on the parental strain analysis, V-Phaser 2 did predict 5 insertions in MiSeq data (Table 4) and 2 insertions in the 24-mix data. Upon further inspection, it appears that all these variants seem to be existing in the input reads. The LPVs in MiSeq data (see Table 4) showed no strand bias while falling in the scope of frequencies of real variants. Since all these cases are present in the homopolymer region (the inserted As are part of a stretch of six As and the G is present in a stretch of eight Gs), these variants could have been artificially introduced during the PCR process. The two insertions in the 24-mix data form a more interesting case, where a one base insertion A is present after position 4135 with respect to the reference and a two base insertion GT is present downstream after position 4320. These two insertions are in phase and are present at the frequency of 0.3342%. As a result, when compared to the normal open reading frame, an additional codon is inserted. Since it does not result in the introduction of any stop codon, the resulting protein is likely functional.

**Table 4:** Characterizing falsely inferred LPVs in 8-mix Illumina MiSeq data

**Reference**	**Inferred LPV**	**Consensus**	**LPV**	**Strand bias**
**position**	**count (+, -)**	**count (+, -)**	**frequency**	***p*-value**
4115	IA (86, 86)	(2131, 2013)	4.15%	0.72
5172	IA (12, 20)	(1632, 2149)	0.85%	0.52
6203	IA (64, 40)	(2494, 2123)	2.25%	0.13
8294	IG (21, 14)	(1923, 1625)	0.99%	0.49
9063	IA (22, 16)	(2054, 2074)	0.92%	0.35

(+, -) denote the positive and negative strands. ‘I’ denotes the LPV is an insertion.

## Conclusions

At a higher sequencing depth of intra-host viral population data, ranging anywhere from a thousand to tens of thousands fold, it is expected that we would pick up signals that were previously unseen at a lower coverage. As such practice becomes routine, we are facing both the computational challenge of controlling run time and memory usage as well as the biological challenge of teasing out real variants from systematic errors.

We have implemented V-Phaser 2 to address these challenges. It overcomes the major performance bottleneck in the previous version and has a better control for false positive predictions. Moreover, V-Phaser 2 has a clear model for length polymorphic variants, which is particularly relevant for the study of chronic diseases like HIV. Nonetheless, these variations may occur in an acute disease as well but could have been missed in lower coverage data due to a lack of power to detect such variants. We believe that V-Phaser 2 would be useful in studying these cases.

A remaining challenge is to improve filtering techniques to further reduce the number of false positives while retaining high sensitivity. Certain systematic errors of unknown origin remain that are called as true variants under the current models and not filtered out by strand bias testing. On the other hand, certain true variants appear to be filtered out by the strand bias filter for reasons that are not well understood. In spite of these challenges, V-Phaser 2 represents a major step forward in our ability to accurately call low level intra-host variation in very deep coverage sequencing data from multiple platforms.

## Competing interests

The authors declare that they have no competing interests.

## Authors’ contributions

XY, MCZ & AM conceived and designed the algorithm; XY implemented V-Phaser 2; XY, PC, AM, MRH & MCZ analyzed data; XY & MCZ wrote the manuscript with input from all authors; All authors read and approved the final manuscript.
